# Revisiting Socransky’s Complexes: A Review Suggesting Updated New Bacterial Clusters (GF-MoR Complexes) for Periodontal and Peri-Implant Diseases and Conditions

**DOI:** 10.3390/microorganisms12112214

**Published:** 2024-10-31

**Authors:** Gustavo Vicentis Oliveira Fernandes, Grace Anne Mosley, William Ross, Ally Dagher, Bruno Gomes dos Santos Martins, Juliana Campos Hasse Fernandes

**Affiliations:** 1 Missouri School of Dentistry & Oral Health, A. T. Still University, 1500 Park Ave, St. Louis, MO 63104, USA; 2 Private Practice, Plymouth, MI 48170, USA; 3 Department of Surgery, University of Salamanca, 37008 Salamanca, Spain; 4 Private Researcher, St. Louis, MO 63104, USA

**Keywords:** bacteria, bacterium, complexes, clusters, periodontitis, peri-implantitis, peri-implant mucositis, gingivitis, periodontal health, Socransky’s complexes, newly identified periodontal pathogens, newly identified peri-implant pathogens

## Abstract

This review aimed to identify newly discovered bacteria from individuals with periodontal/peri-implant diseases and organize them into new clusters (GF-MoR complexes) to update Socransky’s complexes (1998). For methodological development, the PCC (Population, Concept, Context) strategy was used for the focus question construction: “In patients with periodontal and/or peri-implant disease, what bacteria (microorganisms) were detected through laboratory assays?” The search strategy was applied to PubMed/MEDLINE, PubMed Central, and Embase. The search key terms, combined with Boolean markers, were (1) bacteria, (2) microbiome, (3) microorganisms, (4) biofilm, (5) niche, (6) native bacteria, (7) gingivitis), (8) periodontitis, (9) peri-implant mucositis, and (10) peri-implantitis. The search was restricted to the period 1998–2024 and the English language. The bacteria groups in the oral cavity obtained/found were retrieved and included in the GF-MoR complexes, which were based on the disease/condition, presenting six groups: (1) health, (2) gingivitis, (3) peri-implant mucositis, (4) periodontitis, (5) peri-implantitis, and (6) necrotizing and molar–incisor (M-O) pattern periodontitis. The percentual found per group refers to the number of times a specific bacterium was found to be associated with a particular disease. A total of 381 articles were found: 162 articles were eligible for full-text reading (k = 0.92). Of these articles, nine were excluded with justification, and 153 were included in this review (k = 0.98). Most of the studies reported results for the health condition, periodontitis, and peri-implantitis (3 out of 6 GF-MoR clusters), limiting the number of bacteria found in the other groups. Therefore, it became essential to understand that bacterial colonization is a dynamic process, and the bacteria present in one group could also be present in others, such as those observed with the bacteria found in all groups (*Porphyromonas gingivalis*, *Tannarela forsythia*, *Treponema denticola*, and *Aggregatibacter actinomycetemcomitans*) (GF-MoR’s red triangle). The second most observed bacteria were grouped in GF-MoR’s blue triangle: *Porphyromonas* spp., *Prevotela* spp., and *Treponema* spp., which were present in five of the six groups. The third most detected bacteria were clustered in the grey polygon (GF-MoR’s grey polygon): *Fusobacterium nucleatum*, *Prevotella intermedia*, *Campylobacter rectus*, and *Eikenella corrodens*. These three geometric shapes had the most relevant bacteria to periodontal and peri-implant diseases. Specifically, per group, GF-MoR’s health group had 58 species; GF-MoR’s gingivitis group presented 16 bacteria; GF-MoR’s peri-implant mucositis included 17 bacteria; GF-MoR’s periodontitis group had 101 different bacteria; GF-MoR’s peri-implantitis presented 61 bacteria; and the last group was a combination of necrotizing diseases and molar–incisor (M-I) pattern periodontitis, with seven bacteria. After observing the top seven bacteria of all groups, all of them were found to be gram-negative. Groups 4 and 5 (periodontitis and peri-implantitis) presented the same top seven bacteria. For the first time in the literature, GF-MoR’s complexes were presented, gathering bacteria data according to the condition found and including more bacteria than in Socransky’s complexes. Based on this understanding, this study could drive future research into treatment options for periodontal and peri-implant diseases, guiding future studies and collaborations to prevent and worsen systemic conditions. Moreover, it permits the debate about the evolution of bacterial clusters.

## 1. Introduction

In the human oral cavity’s damp and organically rich environment, it is no surprise for many to understand that one’s mouth is an ecosystem where an immense variety of microorganisms endure and thrive. Considering the lifestyle and genomic differences of all individuals, every person can host different types of organisms in their oral cavity, including bacteria, fungi, viruses, and even protozoa. However, a standard balance of predominant species is found in most healthy human beings. Unfortunately, there is currently a lack of convincing in vivo studies that determine precisely what roles fungi and protozoa play in maintaining a healthy, symbiotic environment in the host’s oral cavity, leaving most studies focusing on the most abundant organism in the oral cavity: bacteria [1].

A review published in 2018 aimed to research the expanded Human Oral Microbiome Database (eHOMD) to determine which of the cultivable and uncultivable bacteria found in the oral cavity are indicative of health [2]. With the advantage of 16S rDNA profiling, six broad phyla were found to inhabit a healthy oral cavity, and they constituted 96% of the oral bacteria present in the mouth, including *Firmicutes*, *Actinobacteria*, *Proteobacteria*, *Fusobacteria*, *Bacteroidetes*, and *Spirochaetes*. A relatively steady balance of these incalculable colonies in terms of quantity and quality maintains equilibrium for a stable and, therefore, healthy oral cavity for the host.

Within the field of periodontology, most current research is focused on its four most prevalent diseases: gingivitis, periodontitis, peri-implant mucositis, and peri-implantitis. Periodontal disease is estimated to affect between 45 and 50% of the population of the globe, where 11.2% of them were diagnosed with severe periodontitis [3]. It refers to an infection of the tissues surrounding and supporting the teeth. The collection of bacteria on teeth and gingiva forms a biofilm. Failure in the removal of biofilm/plaque through regular hygiene (brushing and flossing) can allow the slim to harden into calculus, requiring removal only from a dentist (professional care). Early stages of periodontal disease are called gingivitis; it is an inflammation (including, but not limited to, redness, swelling, and profuse bleeding on probing [BoP]) of the gums induced, most of the time, by dental plaque. However, gingivitis can also be caused by certain drugs, stress, malnutrition, genetic and acquired diseases, viral and fungal infections, allergies, and trauma. If gingivitis is not correctly treated or resolved, it may, but not necessarily, evolve into a severe form of the disease known as periodontitis [4]. Periodontitis is the advanced inflammation of the tissues of attachment for dentition (including the alveolar bone, cementum, and periodontal ligaments), marked by the irreversible loss of bone tissue. All of these processes happen due to the dysbiosis process; over time, the periodontal apparatus’s destruction can occur, potentially leading to tooth loss [5].

**Figure 1:**
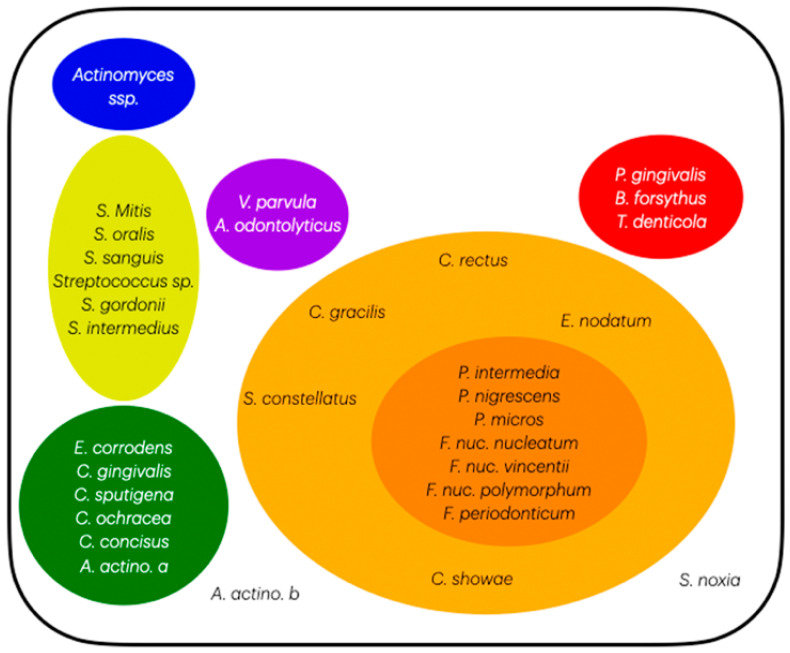
Socransky’s complexes with adaptation, including the blue complex.

The subgingival periodontal microbiome has been a heavily studied topic. In 1998, Socransky et al.’s research [6] identified many microorganisms and didactyly divided them into clusters (Figure 1), becoming an essential aspect of education, research, and treatments. Understanding a specific bacteria’s role in periodontal diseases has guided the evolution of research in microbiological diagnosis and treatment strategies [7,8], targeting interventions to prevent and treat periodontitis effectively.

Socransky’s bacterial complexes in periodontics are essential for understanding the elemental microbiological composition in relation to oral health and periodontal disease through a framework that categorizes the periodontal bacteria into different complexes based on their pathogenicity. According to Socransky et al. [6], microorganisms inhabiting the oral cavity consist of 5 major complexes organized by the severity of each microorganism. Although this research established a profound understanding and framework of the microorganisms related to periodontal diseases, this information is outdated. It poses a vital need to update the bacteria and microorganisms related to developing periodontal and peri-implant diseases. In addition, since the article was published in 1998 [6], there has been no robust collective article suggesting the identification and updates of newly found bacteria that have been recognized through more recent research. Identifying specific microorganisms within one’s subgingival microbiome has been crucial in understanding the pathogenesis of periodontal and peri-implant diseases.

Similar to periodontal diseases’ effect on teeth, peri-implant diseases affect the tissues around a dental implant. Peri-implant mucositis affects the soft tissue, whereas hard and soft tissues are affected in peri-implantitis [9,10,11]. Moreover, these insights about microorganisms have driven the development of diagnostic tools, influenced treatment protocols, and informed preventive strategies. Currently, peri-implant disease development has been considered broadly relevant in terms of understanding the relationship between one’s oral microbiome and disease onset and progression. A study analyzing the prevalence of pre-peri-implantitis and peri-implantitis determined that 31.3% of the patients had pre-peri-implantitis and 56.6% had peri-implantitis, whereas 31.7% were affected by pre-peri-implantitis and 27.9% were affected by peri-implantitis at the implant level [12]. The results indicate that a high number of patients with implants experienced early and advanced inflammation around the dental implants.

Peri-implantitis development leads the patient to poor clinical outcomes, such as bleeding on probing (BoP), progressive marginal bone loss (MBL), purulent secretion, exacerbated bone remodeling, and, in severe cases, the need for explanation as a last resort available [13]. As a result, understanding the relevant microorganisms present in periodontal/peri-implant diseases is a key factor for prevention and treatment; likewise, understanding the pathogenesis of the disease is vital for understanding dysbiosis, homeostasis, allostasis, and when the relationship between microbiota and host becomes detrimental to the host [14]. Recent literature has highlighted the similarities in treatment approaches for periodontal and peri-implant diseases. Antibiotic therapy remains a cornerstone in the management of both conditions. Systemic and local antibiotics have been shown to enhance clinical outcomes by targeting the pathogenic bacteria involved in these inflammatory processes. Patients with a history of periodontitis are at an increased risk for peri-implant diseases, suggesting that antibiotic prophylaxis may be beneficial in these cases to prevent the onset of peri-implantitis [15]. Furthermore, the application of local antibiotics, such as minocycline or doxycycline, has been reported to improve clinical parameters in both periodontal and peri-implant treatments, directly targeting the biofilm associated with these diseases [16].

Laser therapy has also emerged [17,18] as a promising adjunctive treatment modality for both periodontal and peri-implant diseases. It can effectively reduce bacterial load and promote healing in inflamed tissues. A retrospective study indicated that photodynamic therapy using 5-aminolevulinic acid can effectively manage both periodontitis and peri-implantitis, highlighting the potential of laser applications in these conditions [19]. The ability of lasers to selectively target diseased tissue while preserving healthy structures makes them a valuable tool in the treatment arsenal for both diseases. Moreover, the underlying pathophysiological mechanisms of periodontal and peri-implant diseases share significant similarities, further supporting analogous treatment strategies. Both conditions involve a dysbiotic microbial community, which is being studied here, triggering an inflammatory response and leading to tissue destruction. This interconnectedness suggests that effective treatments for one condition may also benefit the other condition.

Thus, after observing the backgrounds of the periodontal and peri-implant conditions and diseases and how outdated the last complexes published are, it was decided that the goal of this review is to identify newly discovered bacteria from individuals with periodontal/peri-implant diseases, thus organizing them into new clusters (GF-MoR complexes) to update Socransky’s complexes. This aim is essential in advancing the knowledge regarding periodontal/peri-implant diseases [20,21] to help clinicians identify bacterial clusters correlated to the disease’s specificity. Mapping and identifying the microorganisms and specific bacteria in the mouth enable professionals to understand the diseases better and create personalized treatment plans; additionally, since oral bacteria can be linked to systemic health issues [22,23], knowing the specific species can help clinicians anticipate potential systemic diseases in patients and refer them to the appropriate specialists.

## 2. Materials and Methods

This review deployed a systematic methodology to better organize the findings and keep the results transparent. The PCC (Population, Concept, Context) strategy was used for the construction of the focus question: “In patients with periodontal and/or peri-implant disease, what bacteria (microorganisms) were detected through laboratory assays?”

### 2.1. Search Strategy

The bibliographic search used three databases: PubMed/MEDLINE, PubMed Central, and Embase. The search process used specific keywords with a combination of Boolean markers (AND, OR): (1) bacteria, (2) microbiome, (3) microorganisms, (4) biofilm, (5) niche, (6) native bacteria, (7) gingivitis, (8) periodontitis, (9) peri-implant mucositis, and (10) peri-implantitis. In addition, the search was restricted to the period 1998–2024 (1998 was chosen because it was the publication date of the last complexes [Socransky’s complexes]) and to English language articles.

### 2.2. Study Selection and Eligibility Criteria

The search and studies selection were independently developed by two investigators (G.M. and W.R.); a third author (G.V.O.F) helped to tie-break in case of disagreement. The inclusion criteria were (1) human studies, (2) patients ≥ 18 years old, (3) cohort, cross-sectional, comparative, and case-control studies, clinical trials, and randomized controlled clinical trials (RCTs), (4) studies that assessed microorganisms in the oral cavity and correlate them with the diagnosis, (5) publications released between 1998 and 2024, and (6) publications released in the English language. The exclusion criteria were (1) in vivo (animals) or only partially in vitro studies, (2) any type of review, editorial letters, or case reports, and (3) any lack of information about the bacteria and diagnosis found.

### 2.3. Data Extraction

The necessary information was retrieved from the included articles and inserted in a spreadsheet (title, authors, and bacteria found per condition/disease). This step was performed by two authors (G.M. and W.R.). Specifically, all the bacteria groups in the oral cavity obtained/found were retrieved.

### 2.4. Socransky’s Complexes

Figure 1 shows the complexes published in 1998 [6]. Socransky’s blue complex is an adaptation for better organization; originally, five complexes were described: green, yellow, purple, orange, and red. This last complex plays a significant role in the pathogenesis of periodontal diseases, such as gingivitis and periodontitis, and is known for its association with severe periodontitis, including *Bacteroides forsythias (Tannarella forsythia)*, *Porphyromonas gingivalis*, and *Treponema denticola* [24,25,26]. It is strictly correlated with periodontally-diseased sites and is the most common bacteria and key pathogen in periodontal disease progression [24,26]. The presence of these pathogens is correlated to dysbiotic changes in the oral microbiome, which can lead to increased inflammation and tissue destruction [24,27,28].

The other complexes are linked to the native microbiota dysbiotic process and the development of gingivitis and later periodontitis [29,30]. Specifically, the orange complex is considered the precedent of the red complex for colonization and proliferation. It consists of *Fusobacterium nucleatum*, *Prevotella intermedia*, *Prevotella nigrescens*, *Peptostreptococcus micros* [8,31], *Streptococcus constellatus*, *Eubacterium nodatum*, *Campylobacter showae*, *Campylobacter gracilis*, and *Campylobacter rectus*. The yellow complex comprises several streptococcus species: *Streptococcus sanguis*, *Streptococcus oralis*, *Streptococcus mitis*, *Streptococcus gordonii*, and *Streptococcus intermedius*. The green complex includes three *Capnocytophaga* spp., *Campylobacter concisus*, *Eikenella corrodens*, and *Aggregatibacter actinomycetemcomitans (serotype a)*. The fifth complex (purple) incorporates *Veillonella parvula* and *Actinomyces odontolyticus*, whereas the blue complex (adaptation of the article for a better organization) includes *Actinomyces* spp.

### 2.5. GF-MoR’s Complexes Organization

The bacteria retrieved from each included study and associated with disease/condition were registered for the GF-MoR group development. The GF-MoR complexes were based on the disease/condition, permitting to gather bacteria in six groups: (1) health, (2) gingivitis, (3) peri-implant mucositis, (4) periodontitis, (5) peri-implantitis, and (6) necrotizing and molar–incisor (M-O) pattern periodontitis. The percentual found refers to the number of times a specific bacterium is associated with a particular disease; hence, a percentual number was calculated to sort them from the most common to the least common bacteria. It is worth reporting that bacteria from healthy patients could be present in any other complex and vice-versa, even though the bacteria were not perceptually found in the included studies.

## 3. Results

**Figure 2:**
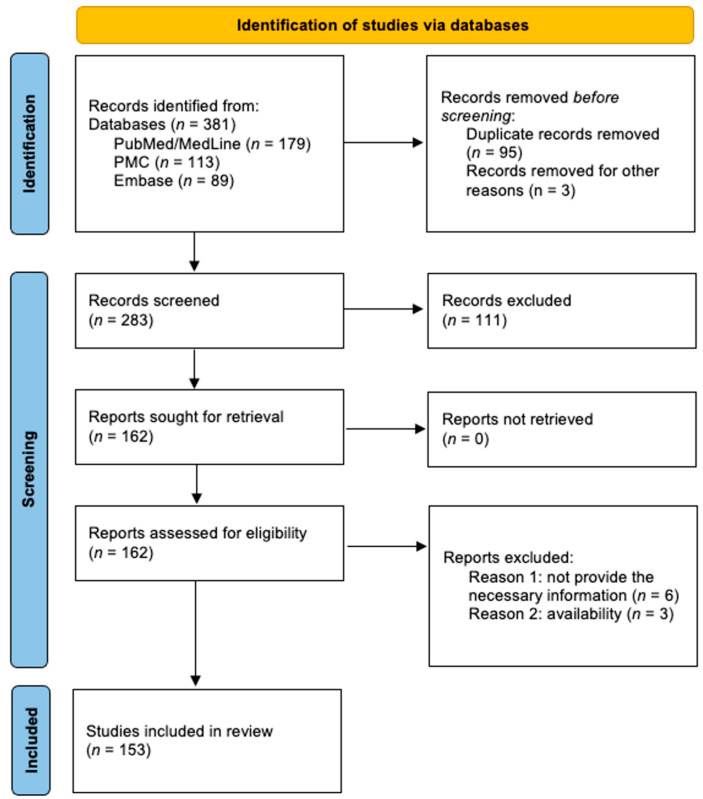
Flowchart for screening and selection of studies.

The screening process resulted in 381 articles. They were first evaluated by their titles and abstracts, and duplicates were removed, resulting in 162 eligible articles for full-text reading (k = 0.92). In this step, nine articles were excluded with justification: six lacked information, and three were excluded because of availability. Hence, 153 articles were included (k = 0.98) (Figure 2, Supplementary Table S1).

**Figure 3:**
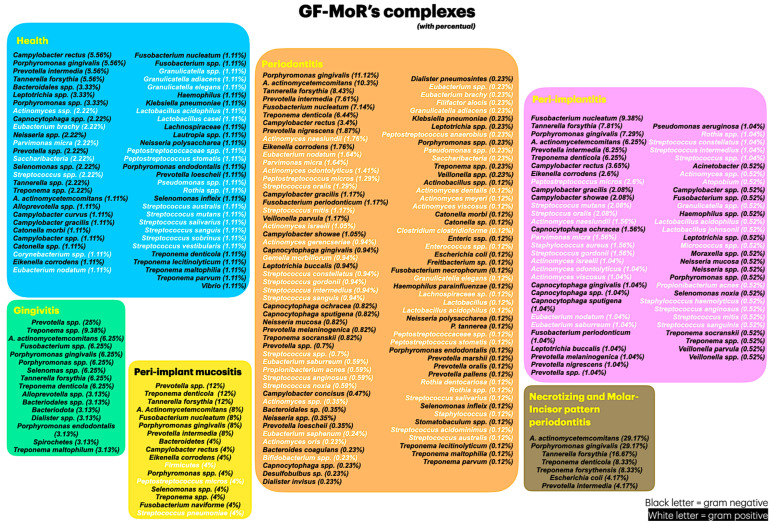
Bacterial organization according to the percentage of citations per condition. (Blue = healthy condition; green = gingivitis; yellow = peri-implant mucositis; orange = periodontitis; brown = necrotizing and M-I pattern periodontitis; and purple = peri-implantitis).

**Figure 4:**
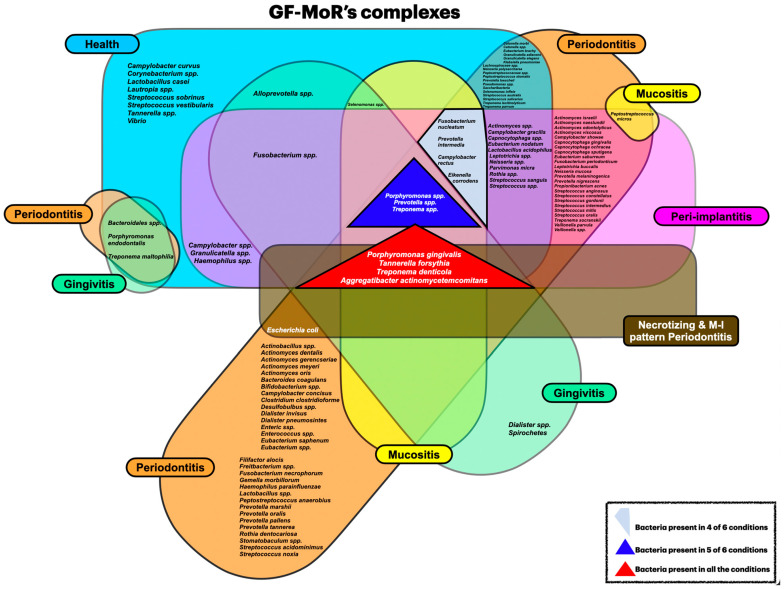
GF-MoR’s complexes organized according to bacteria presence in different clusters. (Blue = healthy condition; green = gingivitis; yellow = peri-implant mucositis; orange = periodontitis; brown = necrotizing and M-I pattern periodontitis; and purple = peri-implantitis).

The majority of the included studies reported results for health, periodontitis, and peri-implantitis conditions (three out of six GF-MoR clusters) (Figure 3); this fact limited the number of bacteria found in the other groups. Therefore, it became essential to understand that bacterial colonization is a dynamic process; bacteria present in one group could also be present in other groups, such as those observed with the bacteria found in all groups (*Porphyromonas gingivalis*, *Tannarela forsythia*, *Treponema denticola*, and *Aggregatibacter actinomycetemcomitans*) (GF-MoR’s red triangle), independent of the condition (Figure 4); the second most observed bacteria were *Porphyromonas* spp., *Prevotela* spp., and *Treponema* spp., which was present in five of the six GF-MoR groups (GF-MoR’s blue triangle). The third most detected bacteria were clustered in the grey polygon (GF-MoR’s polygon) (*Fusobacterium nucleatum*, *Prevotella intermedia*, *Campylobacter rectus*, and *Eikenella corrodens*). These three geometric shapes (not limited to them) had the most relevant bacteria for periodontal and peri-implant diseases.

### 3.1. Clusters and the Seven Most Relevant Bacteria per Group

In the health cluster (Cluster 1), 36 g-negative bacteria and 22 g-positive bacteria were found (totaling 58 species); the top seven most common bacteria found in this group were all gram-negative: 1. *Campylobacter rectus* (5.56%), 2. *Porphyromonas gingivalis* (5.56%), 3. *Prevotella intermedia* (5.56%), 4. *Tannarela forsythia* (5.56%), 5. *Bacteroidales* spp. (3.33%), 6. *Leptotrichia* spp. (3.33%), and 7. *Porphyromonas* spp. (3.33%).

In gingivitis (Cluster 2), 16 bacteria were retrieved, all of which were gram-negative bacteria. The seven most relevant bacteria were 1. *Prevotella* spp. (25%), 2. *Treponema* spp. (9.38%), 3. *Aggregatibacter actinomycetemcomitans* (6.25%), 4. *Fusobacterium* spp. (6.25%), 5. *Porphyromonas gingivalis* (6.25%), 6. *Porphyromonas* spp. (6.25%), and 7. *Selenomas* spp. (6.25%).

In the peri-implant mucositis cluster (Cluster 3), 17 bacteria were detected (3 g-positive and 14 g-negative). The seven most significant bacteria (all gram-negative) were 1. *Prevotella* spp. (12%), 2. *Treponema denticola* (12%), 3. *Tannarela forsythia* (12%), 4. *Aggregatibacter actinomycetemcomitans* (8%), 5. *Fusobacterium nucleatum* (8%), 6. *Porphyromonas gingivalis* (8%), and 7. *Prevotella intermedia* (8%).

In the periodontitis group (Cluster 4), there were 54 g-negative and 47 g-positive bacteria, totaling 101 different bacteria. Again, the seven most significant bacteria were all gram-negative: 1. *Porphyromonas gingivalis* (11.12%), 2. *Aggregatibacter actinomycetemcomitans* (10.3%), 3. *Tannarela forsythia* (8.43%), 4. *Prevotella intermedia* (7.61%), 5. *Fusobacterium nucleatum* (7.14%), 6. *Treponema denticola* (6.44%), and 7. *Campylobacter rectus* (3.4%).

In peri-implantitis (Cluster 5), 34 g-negative and 27 g-positive bacteria were gathered in this group (61 bacteria). The seven most significant bacteria were all gram-negative and the same as the periodontitis group, presenting a similar standard of bacteria: 1. *Fusobacterium nucleatum* (9.38%), 2. *Tannarela forsythia* (7.81%), 3. *Porphyromonas gingivalis* (7.29%), 4. *Aggregatibacter actinomycetemcomitans* (6.25%), 5. *Prevotella intermedia* (6.25%), 6. *Treponema denticola* (6.25%), and 7. *Campylobacter rectus* (3.65%).

The last cluster is a combination of necrotizing diseases and molar–incisor (M-I) pattern periodontitis (Cluster 6); the seven most important were 1. *Aggregatibacter actinomycetemcomitans* (29.17%), 2. *Porphyromonas gingivalis* (29.17%), 3. *Tannarela forsythia* (16.67%), 4. *Treponema denticola* (8.33%), 5. *Treponema forsythensis* (8.33%)*,* 6. *Escherichia coli* (4.17%), and 7. *Prevotella intermedia* (4.17%).

### 3.2. Socransky’s Complexes and GF-MoR’s Complexes

Socranksky et al. [6] did not gather bacteria strictly according to the diseases and conditions found in the present study; in that study, the scholars evaluated the subgingival bacteria in healthy patients and in patients with periodontitis. Moreover, through correspondence analysis, they achieved community ordination, reinforcing and showing the relationships among the complexes. All the bacteria detected by Socransky et al. were also found and retrieved from the literature published after Socransky et al.’s study. They compounded the GF-MoR’s groups, which were divided by diseases and conditions featuring many other bacteria.

**Figure 5:**
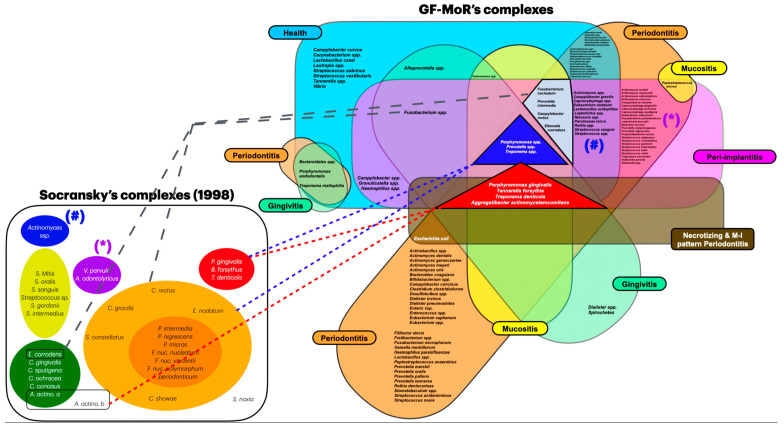
Bacteria allocation in Socransky’s and GF-MoR’s complexes. ([#] Socransky’s blue complex is an adaptation for better organization; originally, five complexes were described: green, purple, yellow, orange, and red). ((*) and (#) are the correlations between Socransky’s and GF-MoR complexes).

The Socransky’s red complex (*P. gingivalis*, *B. forsythus*, and *T. denticola*), which is wholly part of the GF-MoR’s red triangle, was closely associated with the orange complex (*F. nucleatum*, *P. intermedia*, *P. nigrescens*, *P. micros*, *E. nodatum*, *S. constellatus*, and *Campylobacter* spp.); *F. nucleatum*, *P. intermedia*, and *Campylobacter rectus* were included in the GF-MoR’s grey polygon (the third most relevant GF-MoR group). In Socransky et al.’s article, the authors also showed the relationships among the *Capnocytophaga* spp. Most of the *Streptococcus* spp. (Socransky’s yellow complex), *E. corrodens* (present in the GF-MoR’s Polygon) and *C. concisus* (green complex) were closely related and somewhat associated with the orange complex. The *A. odontolyticus* and *V. parvula* relationship (Socransky’s purple complex) was confirmed by cluster and correspondence analyses [6]; both were also found in the intersection of the GF-MoR’s periodontitis and peri-implantitis groups. Furthermore, the authors reinforced the distinction between Socransky’s red and orange clusters and the separation of the red and orange complexes from the other two groups, which were the green and yellow complexes (Figure 5).

## 4. Discussion

This review aimed to identify newly discovered bacteria from individuals with periodontal/peri-implant diseases and conditions, which were clustered in newly updated complexes (GF-MoR complexes). Socransky’s complexes, introduced in 1998 [6], categorized oral bacteria based on the subgingival sites of periodontal disease (periodontitis) and healthy conditions, including patients with and without periodontitis. The present study included not only the periodontitis and health groups but also the gingivitis, peri-implant mucositis, and peri-implantitis groups.

Thus, knowing the types of bacteria in the periodontal and peri-implant conditions aids clinicians in determining the stage of the disease and choosing the most effective treatment. Understanding the bacteria linked to healthy, gingivitis, peri-implant mucositis, and more advanced conditions, such as periodontitis and peri-implantitis, can help create a more accurate diagnosis and aid in treatment planning, especially in cases where there is a fine line/borderline between conditions such as gingivitis and early-stage periodontitis. In addition, verifying whether newly identified bacteria are symbiotic with other bacteria can increase the knowledge of biofilm formation, bacterial community/relationship, structure, and resilience. This insight can help develop strategies to disrupt harmful biofilms and promote a healthy oral microbiome.

Socransky’s complexes [6] are pivotal in understanding the microbial dynamics contributing to periodontitis. The red, orange, yellow, green, and purple complexes have been identified, each with specific bacterial species that exhibit varying pathogenic potentials. Socransky’s yellow, green, and adapted blue complexes, including *Streptococcus mitis* and *Actinomyces* spp., are generally associated with periodontal health; in comparison, the GF-MoR health group has presented a higher number of bacteria. However, their role in the context of disease is complex, as they can also be found in diseased sites, as observed in GF-MoR’s complexes, albeit in lower proportions compared to the red and orange complex bacteria [32,33]. The presence of these complexes may indicate an attempt by the host to restore a healthy microbial balance, but their efficacy in preventing disease progression remains uncertain.

Socransky’s orange complex, particularly *Fusobacterium nucleatum* (a member of the GF-MoR’s grey Polygon), is frequently identified in periodontal and peri-implant diseases. This bacterium bridges early and late colonizers, promoting the establishment of pathogenic communities [34,35]. Furthermore, the microbial diversity in healthy implants is lower than in peri-implantitis, with gram-negative anaerobic bacteria being predominant [36,37]. This shift in microbial composition is critical for understanding peri-implantitis and its pathogenesis and the importance of oral hygiene maintenance in preventing dysbiosis.

A relevant bacteria found either in aggressive and necrotizing periodontitis and peri-implantitis is *Aggregatibacter actinomycetemcomitans* (A.a.) (Socransky’s green complex; GF-MoR’s red triangle) and *Campylobacter rectus* (Socransky’s orange complex; GF-MoR’s grey polygon), which are also found in periodontal disease [38,39,40]. Aggressive forms of periodontitis present with a phenotypic molar–incisor pattern initiated by *A.a.* and form concomitants with diseases of endodontic origin. In GF-MoR’s complexes, *A.a*. receives a higher level of importance than in Socransky’s complexes due to its presence in all the groups, especially in necrotizing and M-I pattern periodontitis (29.17%). Detecting these bacteria in peri-implant or periodontal sites underscores the need to monitor microbial profiles in patients with dental implants or periodontitis. The absence of treatment for any of the diseases studied allows the evolution of the disease in a non-linear and accelerating pattern.

The most relevant of the Socransky’s complexes, the red complex, includes *Porphyromonas gingivalis*, *Tannerella forsythia*, and *Treponema denticola,* which match with GF-MoR’s red triangle; these bacteria are also present in all GF-MoR’s groups; an additional bacteria is also present in the GF-MoR red triangle, *A.a*. Then, it is possible to affirm that Socransky’s red complex and GF-MoR’s red triangle are highly related to periodontal and peri-implant disease progression [38,41,42].

### 4.1. Periodontal and Peri-Implant Diseases and Conditions

Numerous articles have proven that certain bacteria are involved in different stages of disease, from mild gingivitis to advanced periodontitis. Likewise, one study introduced a visual design for clinicians and researchers to easily understand the advancement of a stable, subgingival, pathogenic bacterial colony in periodontal disease [6]. The most virulent and destructive pathogens (*P. gingivalis*, *T. forsythia*, *T. denticola*—Socransky’s red complex, and GF-MoR’s red triangle that also included *A.a.*), with a recent study that reported the importance of *Parvimonas micra* and *Filifactor alocis* as being indicative of severe inflammation [43], showed that the red complex has high-risk initiators of intracellular damage during the later stages of periodontal tissue damage. In parallel, with the higher level of these bacteria, there are substantial decreases in the periodontitis pockets compared to the healthy sulcus for the strains of *Streptococcus sanguinis* (*sanguis*), *Rothia dentocariosa*, *Veillonella parvula*, *Capnocytophaga sputigena*, and *Prevotella intermedia* [43].

Plaque-induced gingivitis is often considered a precursor to periodontitis, but it is not always followed. Studies indicate that bacterial community diversity increases in the crevicular fluid (in the gingiva) from individuals experiencing bleeding, the most relevant and common symptom of gingivitis [37,44]. This increase in diversity is linked to the presence of pathogenic bacteria, which disrupt the homeostatic balance of the oral microbiome. The dysbiotic state fosters an environment conducive to inflammation as the immune system responds to the heightened bacterial load [27,37]. Furthermore, various cytokines mediate the inflammatory response, which is correlated with clinical inflammatory signs of gingivitis [23,27,37].

Periodontitis represents a more advanced stage of periodontal disease, where the inflammatory response leads to the destruction of periodontal tissues, causing attachment loss [23]. It may be multifactorial, but clinicians and researchers can centralize this disease to disrupt the dynamic, symbiotic relationships of the microflora, allowing certain pathogenic species to prosper and thrive. Such disruptions include significant changes in pH or diet, interactions between bacteria, and the absence of mechanical forces from mastication and brushing, among several other disruptions [45]. Furthermore, research has proven that specific intrinsic and extrinsic factors interact with particular pathogenic bacteria, which can be further discussed. The specific bacterium’s role in periodontitis’ etiology has already been well-documented, with *P. gingivalis* being a prominent pathogen due to its virulence factors that facilitate immune evasion and tissue destruction [27,46].

Peri-implant mucositis and peri-implantitis also involve dysbiotic bacterial communities similar to those observed in periodontal diseases. It is characterized by the inflammation of the soft tissue (mucosa) surrounding the dental implant, which is often seen in patients. The microbial profile in peri-implantitis has been shown to include members of Socransky’s red complex and members of the GF-MoR’s red triangle, indicating a shared pathogenic mechanism with periodontitis [24,28]. These bacteria can lead to a mucosal barrier breakdown, facilitating further bacterial invasion and inflammation [46,47]. The interplay between the host immune response and the bacterial community is critical in determining the outcome of these conditions.

Multiple bacterial species interact synergistically to highlight the polymicrobial nature, exacerbating the inflammatory response and contributing to disease progression [24,48]. This complexity highlights a multifaceted necessity to approach the treatment, targeting the bacterial community and the individual’s immune response. The concept of polymicrobial synergy and dysbiosis (PSD) provides a framework for understanding the interactions between different bacterial species in periodontal diseases. This model suggests that certain bacteria present can enhance the pathogenic potential of others, leading to a more severe inflammatory response [8,24]. This fact justifies the importance of Socransky’s and GF-MoR’s complexes. For instance, the co-occurrence of *P. gingivalis* with other bacteria can amplify the inflammatory response, resulting in more significant tissue destruction [24,48]; for example, bacteria from Socransky’s red complex (also present in the GF-MoR’s red triangle) that are associated with Socransky’s orange complex bacteria (present in the GF-MoR’s grey Polygon), which includes species such as *Fusobacterium nucleatum* and *Prevotella intermedia*, will contribute to periodontal diseases progression. There is a co-aggregation that facilitates their colonization and enhances pathogenicity [38,49,50]. This highlights the importance of considering the entire microbial community rather than focusing on individual pathogens when studying periodontal and peri-implant diseases.

### 4.2. Modifiers

Probing pocket depth (PD), clinical attachment loss (CAL), and alveolar bone loss are more prevalent and severe in smoker patients with uncontrolled diabetes or other systemic diseases that can be linked with the pathogenic bacteria of periodontal/peri-implant disease. For smokers, some authors proved that periodontium is affected by defect chemotaxis and phagocytosis of neutrophils, increasing bacterial colonization and impairing clearance [51,52]. Furthermore, smoking is inversely correlated with IgG antibody serum levels, particularly for some periodontal pathogens [53,54]. *P. gingivalis*, *C. rectus*, and *P. nigrescens*, which are considered periodontal/peri-implant pathogens, thrive in smokers after adjusting important confounding factors [55].

Patients with uncontrolled diabetes (any type) are associated with higher levels of systemic inflammatory biomarkers, many of which are found in periodontal and peri-implant diseases as well. Periodontal and peri-implant treatment lowering one’s HbA1c has been proven to decrease this ongoing inflammatory response in serum and periodontal tissues, respectively. Hence, accentuating the mutual impact of treatment of each disease, or lack thereof, affects the control of the other. Moreover, one study recovering periodontal pathogens in both conditions (diabetic and non-diabetic patients) showed that a significantly increased number of individuals with diabetes harbored *P. gingivalis* [56], further outlining the relationship between the two diseases.

Finally, it must not overlook the impact that periodontal/peri-implant diseases and certain systemic diseases have on one another. Examples include cardiovascular disease, gastrointestinal and colorectal cancer, Alzheimer’s disease, and adverse pregnancy outcomes. One study can link many of these systemic diseases with the pathogens of oral disease as well as their metabolic by-products; however, the evidence that there is a mechanism that demonstrates a “cause and effect” relationship has yet to be identified. Furthermore, recent advancements in microbiome research have utilized metagenomic analyses to explore the complex interactions within the oral microbiota. These studies revealed that the microbial compositions in individuals with periodontal disease are markedly different from those of healthy individuals [57,58,59], with specific bacterial taxa being associated with disease severity [24,48]. Identifying these microbial signatures can aid in developing targeted therapeutic strategies, such as probiotics or antimicrobial treatments [60,61], to restore a healthy microbial balance and mitigate inflammation [24,42].

### 4.3. Advances in Techniques for Bacteria Detection

In the past five years, significant advancements have been made in the field of 16S rRNA sequencing, particularly concerning the identification of bacteria in oral conditions. These developments have primarily focused on improving the resolution of bacterial identification, enhancing sequencing technologies, and refining bioinformatics approaches. One of the notable advancements is the application of full-length 16S rRNA gene sequencing, which has been shown to provide superior resolution to traditional short-read sequencing methods. Matsuo et al. [62] demonstrated that full-length sequencing allows for better discrimination among closely related bacterial taxa, such as *Bifidobacterium* and *Clostridium*, which are relevant in oral microbiota studies. This is crucial for accurately identifying bacterial species that may play a role in oral health and disease.

Moreover, the integration of high-throughput sequencing technologies, such as Oxford Nanopore Technologies, has facilitated the analysis of complex microbial communities. Some authors conducted comparative evaluations of second-generation and third-generation sequencing technologies, highlighting that while both have their advantages, third-generation sequencing can yield longer reads that are beneficial for resolving complex bacterial populations in various samples, including oral samples [63]. This capability is particularly important in oral microbiome research, where diverse bacterial species coexist and interact.

The choice of hypervariable regions for amplification has also been emphasized as a critical factor influencing the outcomes of 16S rRNA sequencing. Heidrich et al. noted that different primer pairs targeting various regions of the 16S rRNA gene could yield contrasting results in microbial profiling, underscoring the need for the careful selection of primers in studies focused on microbiota [64]. This is particularly relevant in clinical settings where the accurate identification of pathogens is essential for effective treatment. In addition to these technological advancements, the development of bioinformatics tools has improved the analysis and interpretation of sequencing data. The introduction of integrated databases, such as 16S-ITGDB, has enhanced the classification of prokaryotic sequences, allowing for the more accurate identification of bacterial species associated with various conditions, including oral health [65]. This is particularly beneficial for understanding the complex interactions within the oral microbiome and their implications for oral health. Lastly, applying 16S rRNA sequencing in clinical specimens has shown promising results in identifying bacterial pathogens linked to oral diseases. Eamsakulrat et al. [66] reported a high diagnostic yield of 16S rRNA testing in clinical specimens, suggesting its potential utility in guiding antimicrobial management in oral infections. This highlights the clinical relevance of 16S rRNA sequencing in improving patient outcomes through targeted therapies.

In summary, even though it was not the study’s goal (report techniques used by the articles included), the advancements in 16S rRNA sequencing have significantly enhanced the ability to identify and characterize bacteria in oral conditions and diseases. Combining full-length sequencing, high-throughput technologies, careful primer selection, and improved bioinformatics tools has collectively contributed to more accurate and reliable bacterial identification in the oral microbiome.

### 4.4. Limitations of the Study

One significant limitation was that the majority of the studies reported results for the health, periodontitis, and peri-implantitis groups (mainly for the last two groups), limiting the number of bacteria detected in the other clusters (gingivitis, mucositis, and necrotizing and M-I pattern periodontitis). Moreover, this review used a systematic methodology to standardize the articles’ inclusion; therefore, it has been recommended that all data presented must be carefully analyzed. In addition, this review did not intend to retrieve and compare techniques used for bacterial analysis, limiting our data for the types of bacteria.

## 5. Conclusions

Within the limitations of this study and for the first time in the literature, GF-MoR’s complexes are presented, gathering the bacteria according to the conditions observed and including more bacteria than Socransky’s complexes. Based on this understanding, this study permits the observation of the significance of a specific bacterium, its interconnection with the condition presented by the patient, and the interrelationship of that bacterium between diseases/healthy conditions. Also, it can drive future research into treatment options for periodontal and peri-implant diseases, guiding future studies and collaborating to prevent and worsen systemic conditions. Moreover, it permits debate about the evolution of the bacterial clusters correlated to periodontal and peri-implant diseases and conditions.

## Data Availability

The raw data supporting the conclusions of this article will be made available by the authors upon request.

## Supplementary Materials

The following supporting information can be downloaded at: https://www.mdpi.com/article/10.3390/microorganisms12112214/s1, Table S1: List of articles included.
