# Supplementary material for: Revisiting Socransky’s Complexes: A Review Suggesting Updated New Bacterial Clusters (GF-MoR Complexes) for Periodontal and Peri-Implant Diseases and Conditions
Source: Microorganisms. 2024 Oct 31;12(11):2214. doi: 10.3390/microorganisms12112214 (PMC11596145; doi:10.3390/microorganisms12112214)
Supplement: Supplementary file 1 [file microorganisms-12-02214-s001.zip › microorganisms-3298090-supplementary.pdf]

Supplementary Table S1. List of articles included.

CODE	Study (Title, Authors, Journal, Year of publication, doi)
7	Effects of a stabilized stannous fluoride dentifrice on clinical, immunomodulatory, and microbial outcomes in a human experimental gingivitis model.<br>Fine N, Barbour A, Kaura K, Kerns KA, Chen D, Trivedi HM, Gomez J, Sabharwal A, McLean JS, Darveau RP, Glogauer M. J Periodontol. 2024;95(5):421-431. doi: 10.1002/JPER.22-0710
8	Omega-3 nanoemulgel in prevention of radiation-induced oral mucositis and its associated effect on microbiome: a randomized clinical trial.<br>Morsy BM, El Domiaty S, Meheissen MAM, Heikal LA, Meheissen MA, Aly NM. BMC Oral Health. 2023;23(1):612. doi: 10.1186/s12903-023-03276-5
10	Effect of scaling and root planing with and without minocycline HCl microspheres on periodontal pathogens and clinical outcomes: A randomized clinical trial.<br>Arnett MC, Chanthavisouk P, Costalonga M, Blue CM, Evans MD, Paulson DR. J Periodontol. 2023;94(9):1133-1145. doi: 10.1002/JPER.23-0002
11	Effect of laser-assisted reconstructive surgical therapy of peri-implantitis on protein biomarkers and bacterial load.<br>Di Gianfilippo R, Wang CW, Xie Y, Kinney J, Sugai J, Giannobile WV, Wang HL. Clin Oral Implants Res. 2023;34(4):393-403. doi: 10.1111/clr.14059
12	A Placebo-Controlled Trial to Evaluate Two Locally Delivered Antibiotic Gels (Piperacillin Plus Tazobactam vs. Doxycycline) in Stage III-IV Periodontitis Patients.<br>Ilyes I, Rusu D, Rădulescu V, Vela O, Boariu MI, Roman A, Surlin P, Kardaras G, Boia S, Chinnici S, Jentsch HFR, Stratul SI. Medicina (Kaunas). 2023;59(2):303. doi: 10.3390/medicina59020303
15	Clinical Evaluation of Diode Laser-Assisted Surgical Periodontal Therapy: A Randomized Split-Mouth Clinical Trial and Bacteriological Study.<br>Doğan ŞB, Akça G. Photobiomodul Photomed Laser Surg. 2022;40(9):646-655. doi: 10.1089/photob.2022.0035
17	Different scaling and root planing strategies in Turkish patients with aggressive periodontitis: A randomized controlled clinical trial.<br>Mamaklıoğlu D, Karched M, Kuru L, Kuru B, Asikainen S, Doğan B. Int J Dent Hyg. 2022;20(2):347-363. doi: 10.1111/idh.12592
19	Chemomechanical preparation influences the microbial community and the levels of LPS, LTA and cytokines in combined endodontic-periodontal lesions: A clinical study.<br>Gomes BPFA, Berber VB, Marinho ACS, Louzada LM, Arruda-Vasconcelos R, Passini MRZ, Lopes EM, Pecorari VGA, Chen T, Paster BJ. J Periodontal Res. 2022;57(2):341-356. doi: 10.1111/jre.12964
21	The microbiome of dental and peri-implant subgingival plaque during peri-implant mucositis therapy: A randomized clinical trial.<br>Philip J, Buijs MJ, Pappalardo VY, Crielaard W, Brandt BW, Zaura E. J Clin Periodontol. 2022;49(1):28-38. doi: 10.1111/jcpe.13566
25	Evaluation of different materials used for sealing of implant abutment access channel and the peri-implant sulcus microbiota: A 6-month, randomized controlled trial.<br>Rubino CV, Katz BG, Langlois K, Wang HH, Carrion JA, Walker SG, Collier JL, Iacono VJ, Myneni SR. Clin Oral Implants Res. 2021;32(8):941-950. doi: 10.1111/clr.13787
28	Effectiveness of single versus multiple sessions of photodynamic therapy as adjunct to scaling and root planing on periodontopathogenic bacteria in patients with periodontitis.<br>Muzaheed, Acharya S, Hakami AR, Allemailem KS, Alqahtani K, Al Saffan A, Aldakheel FM, Divakar DD. Photodiagnosis Photodyn Ther. 2020;32:102035. doi: 10.1016/j.pdpdt.2020.102035
29	Effects on clinical outcomes of adjunctive moxifloxacin versus amoxicillin plus metronidazole in periodontitis patients harboring Aggregatibacter actinomycetemcomitans, Porphyromonas gingivalis, and Tannerella forsythia: exploratory analyses from a clinical trial.<br>Ardila CM, Hernández-Casas C, Bedoya-García JA. Quint Int. 2021;52(1):20-29. doi: 10.3290/j.qi.a44927
34	Clinical and microbiological effect of frequent subgingival air polishing on periodontal conditions: a split-mouth randomized controlled trial.<br>Sekino S, Ogawa T, Murakashi E, Ito H, Numabe Y. Odontology. 2020;108(4):688-696. doi: 10.1007/s10266-020-00493-0
39	Clinical and Microbiological Evaluation of Surgical and Nonsurgical Treatment of Aggressive Periodontitis.<br>Cirino CCDS, Vale HFD, Casati MZ, Sallum EA, Casarin RCV, Sallum AW. Braz Dent J. 2019;30(6):577-586. doi: 10.1590/0103-6440201902930
41	Clinical and microbiological outcomes of photodynamic and systemic antimicrobial therapy in smokers with peri-implant inflammation.<br>Deeb MA, Alsahhaf A, Mubaraki SA, Alhamoudi N, Al-Aali KA, Abduljabbar T. Photodiagnosis Photodyn Ther. 2020;29:101587. doi: 10.1016/j.pdpdt.2019.101587
43	Short-term effects of hyaluronic acid on the subgingival microbiome in peri-implantitis: A randomized controlled clinical trial.<br>Soriano-Lerma A, Magán-Fernández A, Gijón J, Sánchez-Fernández E, Soriano M, García-Salcedo JA, Mesa F. J Periodontol. 2020;91(6):734-745. doi: 10.1002/JPER.19-0184
46	Microbiological dynamics of red complex bacteria following full-mouth air polishing in periodontally healthy subjects-a randomized clinical pilot study.

	Reinhardt B, Klocke A, Neering SH, Selbach S, Peters U, Flemmig TF, Beikler T. Clin Oral Investig. 2019;23(10):3905-3914. doi: 10.1007/s00784-019-02821-3
47	Periodontal condition in Japanese coronary heart disease patients: A comparison between coronary and non-coronary heart diseases.<br>Aoyama N, Kobayashi N, Hanatani T, Ashigaki N, Yoshida A, Shiheido Y, Sato H, Takamura C, Yoshikawa S, Matsuo K, Izumi Y, Isobe M. J Periodontal Res. 2019;54(3):259-265. doi: 10.1111/jre.12626
48	Impact of dental cement on the peri-implant biofilm-microbial comparison of two different cements in an in vivo observational study.<br>Korsch M, Marten SM, Walther W, Vital M, Pieper DH, Dötsch A. Clin Implant Dent Relat Res. 2018;20(5):806-813. doi: 10.1111/cid.12650
50	The Effects of Antimicrobial Peptide Nal-P-113 on Inhibiting Periodontal Pathogens and Improving Periodontal Status.<br>Wang H, Ai L, Zhang Y, Cheng J, Yu H, Li C, Zhang D, Pan Y, Lin L. Biomed Res Int. 2018;2018:1805793. doi: 10.1155/2018/1805793
51	What is the influence of tonsillectomy on the level of periodontal pathogens on the tongue dorsum and in periodontal pockets.<br>Diener VN, Gay A, Soyka MB, Attin T, Schmidlin PR, Sahrmann P. BMC Oral Health. 2018;18(1):62. doi: 10.1186/s12903-018-0521-7
52	Diamond burs versus curettes in root planing: a randomized clinical trial.<br>Türktekin F, Buduneli N, Lappin DF, Türk T, Buduneli E. Aust Dent J. 2018;63(2):242-252. doi: 10.1111/adj.12602
53	Diversity analysis of subgingival microbial bacteria in peri-implantitis in Uygur population.<br>Gao X, Zhou J, Sun X, Li X, Zhou Y. Medicine (Baltimore). 2018;97(5):e9774. doi: 10.1097/MD.0000000000009774
54	Clinical and microbiological evaluation of the effect of Lactobacillus reuteri in the treatment of mucositis and peri-implantitis: A triple-blind randomized clinical trial.<br>Galofré M, Palao D, Vicario M, Nart J, Violant D. J Periodontal Res. 2018;53(3):378-390. doi: 10.1111/jre.12523
56	The effects of Lactobacillus reuteri probiotics combined with azithromycin on peri-implantitis: A randomized placebo-controlled study.<br>Tada H, Masaki C, Tsuka S, Mukaibo T, Kondo Y, Hosokawa R. J Prosthodont Res. 2018;62(1):89-96. doi: 10.1016/j.jpor.2017.06.006
59	Combined application of Er:YAG and Nd:YAG lasers in treatment of chronic periodontitis. A split-mouth, single-blind, randomized controlled trial.<br>Sağlam M, Köseoğlu S, Taşdemir I, Erbak Yılmaz H, Savran L, Sütçü R. J Periodontal Res. 2017;52(5):853-862. doi: 10.1111/jre.12454
61	Bacterial colonization of the peri-implant sulcus in dentate patients: a prospective observational study.<br>Stokman MA, van Winkelhoff AJ, Vissink A, Spijkervet FK, Raghoebar GM. Clin Oral Investig. 2017;21(2):717-724. doi: 10.1007/s00784-016-1941-x
63	Impact of implant-abutment connection on osteoimmunological and microbiological parameters in short implants: a randomized controlled clinical trial.<br>Öztürk VÖ, Emingil G, Bostanci N, Belibasakis GN. Clin Oral Implants Res. 2017;28(9):e111-e120. doi: 10.1111/clr.12937
66	Influence of a triclosan toothpaste on periodontopathic bacteria and periodontitis progression in cardiovascular patients: a randomized controlled trial.<br>Seymour GJ, Palmer JE, Leishman SJ, Do HL, Westerman B, Carle AD, Faddy MJ, West MJ, Cullinan MP. J Periodontal Res. 2017;52(1):61-73. doi: 10.1111/jre.12369
68	Short-term microbiological effects of photodynamic therapy in non-surgical periodontal treatment of residual pockets: A split-mouth RCT.<br>Corrêa MG, Oliveira DH, Saraceni CH, Ribeiro FV, Pimentel SP, Cirano FR, Casarin RC. Lasers Surg Med. 2016;48(10):944-950. doi: 10.1002/lsm.22449
69	Subgingivally applied minocycline microgranules in subjects with chronic periodontitis: A randomized clinical and microbiological trial.<br>Chiappe VB, Gómez MV, Rodríguez C, Fresolone M, Romanelli HJ. Acta Odontol Latinoam. 2015;28(2):122-31. doi: 10.1590/S1852-48342015000200005
70	Clinical and Microbiologic Evaluation of Scaling and Root Planing per Quadrant and One-Stage Full-Mouth Disinfection Associated With Azithromycin or Chlorhexidine: A Clinical Randomized Controlled Trial.<br>Fonseca DC, Cortelli JR, Cortelli SC, Miranda Cota LO, Machado Costa LC, Moreira Castro MV, Oliveira Azevedo AM, Costa FO. J Periodontol. 2015;86(12):1340-51. doi: 10.1902/jop.2015.150227
73	Alcohol Consumption and Periodontitis: Quantification of Periodontal Pathogens and Cytokines.<br>Lages EJ, Costa FO, Cortelli SC, Cortelli JR, Cota LO, Cyrino RM, Lages EM, Nobre-Franco GC, Brito JA, Gomez RS. J Periodontol. 2015;86(9):1058-68. doi: 10.1902/jop.2015.150087
78	Microbiologic Observations After Four Treatment Strategies Among Patients With Periodontitis Maintaining a High Standard of Oral Hygiene: Secondary Analysis of a Randomized Controlled Clinical Trial.<br>Preus HR, Dahlen G, Gjermo P, Baelum V. J Periodontol. 2015;86(7):856-65. doi: 10.1902/jop.2015.140620
84	The effect of metronidazole on the presence of P. gingivalis and T. forsythia at 3 and 12 months after different periodontal treatment strategies evaluated in a randomized, clinical trial.<br>Preus HR, Gjermo P, Scheie AA, Baelum V. Acta Odontol Scand. 2015;73(4):258-66. doi: 10.3109/00016357.2014.920106
88	Clinical, microbial, and immune responses observed in patients with diabetes after treatment for gingivitis: a three-month randomized clinical trial.<br>Raslan SA, Cortelli JR, Costa FO, Aquino DR, Franco GC, Cota LO, Gargioni-Filho A, Cortelli SC. J Periodontol. 2015;86(4):516-26. doi: 10.1902/jop.2014.140197

89	Adjunctive moxifloxacin in the treatment of generalized aggressive periodontitis patients: clinical and microbiological results of a randomized, triple-blind and placebo-controlled clinical trial.<br>Ardila CM, Martelo-Cadavid JF, Boderth-Acosta G, Ariza-Garcés AA, Guzmán IC. J Clin Periodontol. 2015;42(2):160-8. doi: 10.1111/jcpe.12345
92	The association of clinical and microbiologic parameters with histologic observations in relatively healthy peri-implant conditions- a preliminary short-term in vivo study.<br>van Brakel R, Meijer GJ, de Putter C, Verhoeven JW, Jansen J, Cune MS. Int J Prosthodont. 2014;27(6):573-6. doi: 10.11607/ijp.3922
93	Pilot study on the clinical and microbiological effect of subgingival glycine powder air polishing using a cannula-like jet.<br>Kargas K, Tsalikis L, Sakellari D, Menexes G, Konstantinidis A. Int J Dent Hyg. 2015;13(3):161-9. doi: 10.1111/idh.12104
94	Microbial signature profiles of periodontally healthy and diseased patients.<br>Lourenço TG, Heller D, Silva-Boghossian CM, Cotton SL, Paster BJ, Colombo AP. J Clin Periodontol. 2014;41(11):1027-36. doi: 10.1111/jcpe.12302
95	Impact of baseline microbiological status on clinical outcomes in generalized aggressive periodontitis patients treated with or without adjunctive amoxicillin and metronidazole: an exploratory analysis from a randomized controlled clinical trial.<br>Guerrero A, Nibali L, Lambertenghi R, Ready D, Suvan J, Griffiths GS, Wilson M, Tonetti MS. J Clin Periodontol. 2014;41(11):1080-9. doi: 10.1111/jcpe.12299
96	Clinical and microbiological effects of systemic azithromycin in adjunct to nonsurgical periodontal therapy in treatment of Aggregatibacter actinomycetemcomitans associated periodontitis: a randomized placebo-controlled clinical trial.<br>Martande SS, Pradeep AR, Singh SP, Kumari M, Naik SB, Suke DK, Singh P. J Investig Clin Dent. 2016;7(1):72-80. doi: 10.1111/jicd.12115
98	Metronidazole and amoxicillin as adjuncts to scaling and root planing for the treatment of type 2 diabetic subjects with periodontitis: 1-year outcomes of a randomized placebo-controlled clinical trial.<br>Miranda TS, Feres M, Perez-Chaparro PJ, Faveri M, Figueiredo LC, Tamashiro NS, Bastos MF, Duarte PM. J Clin Periodontol. 2014;41(9):890-9. doi: 10.1111/jcpe.12282
99	Internal bacterial colonization of implants: association with peri-implant bone loss.<br>Jervøe-Storm PM, Jepsen S, Jöhren P, Mericske-Stern R, Enkling N. Clin Oral Implants Res. 2015;26(8):957-963. doi: 10.1111/clr.12421
101	Metronidazole alone or with amoxicillin as adjuncts to non-surgical treatment of chronic periodontitis: a secondary analysis of microbiological results from a randomized clinical trial.<br>Soares GM, Mendes JA, Silva MP, Faveri M, Teles R, Socransky SS, Wang X, Figueiredo LC, Feres M. J Clin Periodontol. 2014;41(4):366-76. doi: 10.1111/jcpe.12217
103	Clinical and microbiological effects of levofloxacin in the treatment of chronic periodontitis: a randomized, placebo-controlled clinical trial.<br>Pradeep AR, Singh SP, Martande SS, Naik SB, N P, Kalra N, Suke DK. J Investig Clin Dent. 2015;6(3):170-8. doi: 10.1111/jicd.12091
104	Photodynamic therapy during supportive periodontal care: clinical, microbiologic, immunoinflammatory, and patient-centered performance in a split-mouth randomized clinical trial.<br>Kolbe MF, Ribeiro FV, Luchesi VH, Casarin RC, Sallum EA, Nociti FH Jr, Ambrosano GM, Cirano FR, Pimentel SP, Casati MZ. J Periodontol. 2014;85(8):e277-86. doi: 10.1902/jop.2014.130559
107	Oral prophylaxis and its effects on halitosis-associated and inflammatory parameters in patients with chronic periodontitis.<br>Guentsch A, Pfister W, Cachovan G, Raschke G, Kuepper H, Schaefer O, Eick S. Int J Dent Hyg. 2014;12(3):199-207. doi: 10.1111/idh.12063
109	Oral hygiene reinforcement in the simplified periodontal treatment of 1 hour.<br>Apatzidou DA, Zygogianni P, Sakellari D, Konstantinidis A. J Clin Periodontol. 2014;41(2):149-56. doi: 10.1111/jcpe.12200
110	Photodynamic therapy to treat periimplantitis.<br>Bombeccari GP, Guzzi G, Gualini F, Gualini S, Santoro F, Spadari F. Implant Dent. 2013;22(6):631-8. doi: 10.1097/01.id.0000433592.18679.91
112	Clinical and microbiological effects of Lactobacillus reuteri probiotics in the treatment of chronic periodontitis: a randomized placebo-controlled study.<br>Teughels W, Durukan A, Ozcelik O, Pauwels M, Quirynen M, Haytac MC. J Clin Periodontol. 2013;40(11):1025-35. doi: 10.1111/jcpe.12155
113	The evaluation of enamel matrix derivative on subgingival microbial environment in non-surgical periodontal therapy.<br>Wyganowska-Świątkowska M, Szkaradkiewicz AK, Karpiński TM, Marcinkowski JT. Ann Agric Environ Med. 2013;20(3):431-5.
115	Immunological and microbiological findings after the application of two periodontal surgical techniques: a randomized, controlled clinical trial.<br>Kyriazis T, Gkrizioti S, Tsalikis L, Sakellari D, Deligianidis A, Konstantinidis A. J Clin Periodontol. 2013;40(11):1036-42. doi: 10.1111/jcpe.12149
116	Effects of 2 bracket and ligation types on plaque retention: a quantitative microbiologic analysis with real-time polymerase chain reaction.<br>Baka ZM, Basciftci FA, Arslan U. Am J Orthod Dentofacial Orthop. 2013;144(2):260-7. doi: 10.1016/j.ajodo.2013.03.022
118	LPS-induced inflammatory response after therapy of aggressive periodontitis.

	Shaddox LM, Gonçalves PF, Vovk A, Allin N, Huang H, Hou W, Aukhil I, Wallet SM. J Dent Res. 2013;92(8):702-8. doi: 10.1177/0022034513495242
119	Porphyromonas gingivalis, Treponema denticola and toll-like receptor 2 are associated with hypertensive disorders in placental tissue: a case-control study.<br>Chaparro A, Blanlot C, Ramírez V, Sanz A, Quintero A, Inostroza C, Bittner M, Navarro M, Illanes SE. J Periodontal Res. 2013;48(6):802-9. doi: 10.1111/jre.12074
120	Effect of periodontal therapy on the subgingival microbiota over a 2-year monitoring period. I. Overall effect and kinetics of change.<br>Socransky SS, Haffajee AD, Teles R, Wennstrom JL, Lindhe J, Bogren A, Hasturk H, van Dyke T, Wang X, Goodson JM. J Clin Periodontol. 2013;40(8):771-80. doi: 10.1111/jcpe.12117
122	A randomized controlled clinical trial on the clinical and microbiological efficacy of systemic satranidazole in the treatment of chronic periodontitis.<br>Pradeep AR, Priyanka N, Kalra N, Naik SB. J Int Acad Periodontol. 2013 Apr;15(2):43-50.
125	Clinical and microbiological effects of systemic antimicrobials combined to an anti-infective mechanical debridement for the management of aggressive periodontitis: a 12-month randomized controlled trial.<br>Silva-Senem MX, Heller D, Varela VM, Torres MC, Feres-Filho EJ, Colombo AP. J Clin Periodontol. 2013;40(3):242-51. doi: 10.1111/jcpe.12052
126	Periodontal status and bacteremia with oral viridans streptococci and coagulase negative staphylococci in allogeneic hematopoietic stem cell transplantation recipients: a prospective observational study.<br>Raber-Durlacher JE, Laheij AM, Epstein JB, Epstein M, Geerligs GM, Wolffe GN, Blijlevens NM, Donnelly JP. Support Care Cancer. 2013;21(6):1621-7. doi: 10.1007/s00520-012-1706-2
127	Implant decontamination during surgical peri-implantitis treatment: a randomized, double-blind, placebo-controlled trial.<br>de Waal YC, Raghoebar GM, Huddleston Slater JJ, Meijer HJ, Winkel EG, van Winkelhoff AJ. J Clin Periodontol. 2013;40(2):186-95. doi: 10.1111/jcpe.12034
129	Boric acid irrigation as an adjunct to mechanical periodontal therapy in patients with chronic periodontitis: a randomized clinical trial.<br>Sağlam M, Arslan U, Buket Bozkurt Ş, Hakki SS. J Periodontol. 2013;84(9):1297-308. doi: 10.1902/jop.2012.120467
130	Hyaluronic Acid as an adjunct after scaling and root planing: a prospective randomized clinical trial.<br>Eick S, Renatus A, Heinicke M, Pfister W, Stratul SI, Jentsch H. J Periodontol. 2013;84(7):941-9. doi: 10.1902/jop.2012.120269
132	Influence of IL-6 haplotypes on clinical and inflammatory response in aggressive periodontitis.<br>Nibali L, Pelekos G, D'Aiuto F, Chaudhary N, Habeeb R, Ready D, Parkar M, Donos N. Clin Oral Investig. 2013;17(4):1235-42. doi: 10.1007/s00784-012-0804-3
135	Er:YAG laser in the treatment of periodontal sites with recurring chronic inflammation: a 12-month randomized, controlled clinical trial.<br>Krohn-Dale I, Bøe OE, Enersen M, Leknes KN. J Clin Periodontol. 2012;39(8):745-52. doi: 10.1111/j.1600-051X.2012.01912.x
136	Effects of systemic sitafloxacin on periodontal infection control in elderly patients.<br>Nakajima T, Okui T, Miyauchi S, Honda T, Shimada Y, Ito H, Akazawa K, Yamazaki K. Gerodontology. 2012;29(2):e1024-32. doi: 10.1111/j.1741-2358.2011.00605.x
137	Effect of azithromycin, as an adjunct to nonsurgical periodontal treatment, on microbiological parameters and gingival crevicular fluid biomarkers in generalized aggressive periodontitis.<br>Emingil G, Han B, Ozdemir G, Tervahartiala T, Vural C, Atilla G, Baylas H, Sorsa T. J Periodontal Res. 2012;47(6):729-39. doi: 10.1111/j.1600-0765.2012.01488.x
138	Systemic antibiotics and debridement of peri-implant mucositis. A randomized clinical trial.<br>Hallström H, Persson GR, Lindgren S, Olofsson M, Renvert S. J Clin Periodontol. 2012;39(6):574-81. doi: 10.1111/j.1600-051X.2012.01884.x
139	Clinical and microbiological effects of ozone nano-bubble water irrigation as an adjunct to mechanical subgingival debridement in periodontitis patients in a randomized controlled trial.<br>Hayakumo S, Arakawa S, Mano Y, Izumi Y. Clin Oral Investig. 2013;17(2):379-88. doi: 10.1007/s00784-012-0711-7
140	Clinical and microbiological evaluation of high intensity diode laser adjutant to non-surgical periodontal treatment: a 6-month clinical trial.<br>Euzebio Alves VT, de Andrade AK, Toaliar JM, Conde MC, Zezell DM, Cai S, Pannuti CM, De Micheli G. Clin Oral Investig. 2013;17(1):87-95. doi: 10.1007/s00784-012-0703-7
142	Connective tissue graft plus resin-modified glass ionomer restoration for the treatment of gingival recession associated with non-carious cervical lesions: microbiological and immunological results.<br>Santamaria MP, Casati MZ, Nociti FH Jr, Sallum AW, Sallum EA, Aukhil I, Wallet SM, Shaddox LM. Clin Oral Investig. 2013;17(1):67-77. doi: 10.1007/s00784-012-0690-8
143	Effects of oil drops containing Lactobacillus salivarius WB21 on periodontal health and oral microbiota producing volatile sulfur compounds.<br>Suzuki N, Tanabe K, Takeshita T, Yoneda M, Iwamoto T, Oshiro S, Yamashita Y, Hirofuji T. J Breath Res. 2012;6(1):017106. doi: 10.1088/1752-7155/6/1/017106
144	A randomized clinical trial on the clinical and microbiological efficacy of a xanthan gel with chlorhexidine for subgingival use.<br>Matesanz P, Herrera D, Echeverría A, O'Connor A, González I, Sanz M. Clin Oral Investig. 2013;17(1):55-66. doi: 10.1007/s00784-012-0685-5

145	Azithromycin as an adjunctive treatment of generalized severe chronic periodontitis: clinical, microbiologic, and biochemical parameters.<br>Han B, Emingil G, Özdemir G, Tervahartiala T, Vural C, Atilla G, Baylas H, Sorsa T. J Periodontol. 2012;83(12):1480-91. doi: 10.1902/jop.2012.110519
146	The combination of amoxicillin and metronidazole improves clinical and microbiologic results of one-stage, full-mouth, ultrasonic debridement in aggressive periodontitis treatment.<br>Casarin RC, Peloso Ribeiro ED, Sallum EA, Nociti FH Jr, Gonçalves RB, Casati MZ. J Periodontol. 2012;83(8):988-98. doi: 10.1902/jop.2012.110513
148	Efficacy of locally-delivered doxycycline microspheres in chronic localized periodontitis and on Porphyromonas gingivalis.<br>Rao SK, Setty S, Acharya AB, Thakur SL. J Investig Clin Dent. 2012;3(2):128-34. doi: 10.1111/j.2041-1626.2011.00110.x
149	Effect of a self-etching adhesive containing an antibacterial monomer on clinical periodontal parameters and subgingival microbiologic composition in orthodontic patients.<br>Amasyali M, Enhos S, Uysal T, Saygun I, Kilic A, Bedir O. Am J Orthod Dentofacial Orthop. 2011;140(4):e147-53. doi: 10.1016/j.ajodo.2011.02.022
150	Microbiologic findings 1 year after partial- and full-mouth scaling in the treatment of moderate chronic periodontitis.<br>Knöfler GU, Purschwitz RE, Eick S, Pfister W, Roedel M, Jentsch HF. Quintessence Int. 2011;42(9):e107-17.
153	Clinical and microbiologic results 12 months after scaling and root planing with different irrigation solutions in patients with moderate chronic periodontitis: a pilot randomized trial.<br>Krück C, Eick S, Knöfler GU, Purschwitz RE, Jentsch HF. J Periodontol. 2012;83(3):312-20. doi: 10.1902/jop.2011.110044
157	Microbiologic results after non-surgical erbium-doped:yttrium, aluminum, and garnet laser or air-abrasive treatment of peri-implantitis: a randomized clinical trial.<br>Persson GR, Roos-Jansåker AM, Lindahl C, Renvert S. J Periodontol. 2011;82(9):1267-78. doi: 10.1902/jop.2011.100660
158	Bacterial adhesion and colonization differences between zirconium oxide and titanium alloys: an in vivo human study.<br>Salihoglu U, Boynuegri D, Engin D, Duman AN, Gokalp P, Balos K. Int J Oral Maxillofac Implants. 2011;26(1):101-7.
159	Impact of systemic antimicrobials combined with anti-infective mechanical debridement on the microbiota of generalized aggressive periodontitis: a 6-month RCT.<br>Heller D, Varela VM, Silva-Senem MX, Torres MC, Feres-Filho EJ, Colombo AP. J Clin Periodontol. 2011;38(4):355-64. doi: 10.1111/j.1600-051X.2011.01707.x
161	Comparison of gingival crevicular fluid sampling methods in patients with severe chronic periodontitis.<br>Guentsch A, Kramesberger M, Sroka A, Pfister W, Potempa J, Eick S. J Periodontol. 2011;82(7):1051-60. doi: 10.1902/jop.2011.100565
162	Early bacterial colonization and soft tissue health around zirconia and titanium abutments: an in vivo study in man.<br>van Brakel R, Cune MS, van Winkelhoff AJ, de Putter C, Verhoeven JW, van der Reijden W. Clin Oral Implants Res. 2011;22(6):571-7. doi: 10.1111/j.1600-0501.2010.02005.x
164	Efficacy and safety of adjunctive local moxifloxacin delivery in the treatment of periodontitis.<br>Flemmig TF, Petersilka G, Völp A, Gravemeier M, Zilly M, Mross D, Prior K, Yamamoto J, Beikler T. J Periodontol. 2011;82(1):96-105. doi: 10.1902/jop.2010.100124
167	Nd:YAG (1064 nm) laser for the treatment of chronic periodontitis: a pilot study.<br>Jensen J, Lulic M, Heitz-Mayfield LJ, Joss A, Lang NP. J Investig Clin Dent. 2010;1(1):16-22. doi: 10.1111/j.2041-1626.2010.00009.x
172	Mechanical non-surgical treatment of peri-implantitis: a single-blinded randomized longitudinal clinical study. II. Microbiological results.<br>Persson GR, Samuelsson E, Lindahl C, Renvert S. J Clin Periodontol. 2010;37(6):563-73. doi: 10.1111/j.1600-051X.2010.01561.x
173	The recolonization hypothesis in a full-mouth or multiple-session treatment protocol: a blinded, randomized clinical trial.<br>Zijnge V, Meijer HF, Lie MA, Tromp JA, Degener JE, Harmsen HJ, Abbas F. J Clin Periodontol. 2010;37(6):518-25. doi: 10.1111/j.1600-051X.2010.01562.x
175	Clinical and microbiologic follow-up evaluations after non-surgical periodontal treatment with erbium:YAG laser and scaling and root planing.<br>Lopes BM, Theodoro LH, Melo RF, Thompson GM, Marcantonio RA. J Periodontol. 2010;81(5):682-91. doi: 10.1902/jop.2010.090300
176	Full-mouth antimicrobial photodynamic therapy in Fusobacterium nucleatum-infected periodontitis patients.<br>Sigusch BW, Engelbrecht M, Völpel A, Holletschke A, Pfister W, Schütze J. J Periodontol. 2010;81(7):975-81. doi: 10.1902/jop.2010.090246
178	Efficacy of amoxicillin and metronidazole combination for the management of generalized aggressive periodontitis.<br>Yek EC, Cintan S, Topcuoglu N, Kulekci G, Issever H, Kantarci A. J Periodontol. 2010;81(7):964-74. doi: 10.1902/jop.2010.090522
179	Microbiologic testing and outcomes of full-mouth scaling and root planing with or without amoxicillin/metronidazole in chronic periodontitis.<br>Cionca N, Giannopoulou C, Ugolotti G, Mombelli A. J Periodontol. 2010;81(1):15-23. doi: 10.1902/jop.2009.090390
180	Photodynamic therapy of persistent pockets in maintenance patients-a clinical study.<br>Rühling A, Fanghänel J, Houshmand M, Kuhr A, Meisel P, Schwahn C, Kocher T. Clin Oral Investig. 2010;14(6):637-44. doi: 10.1007/s00784-009-0347-4
183	One-stage full-mouth versus partial-mouth scaling and root planing during the effective half-life of systemically administered azithromycin.

	Yashima A, Gomi K, Maeda N, Arai T. J Periodontol. 2009;80(9):1406-13. doi: 10.1902/jop.2009.090067
184	Clinical and microbiological benefits of strict supragingival plaque control as part of the active phase of periodontal therapy.<br>Feres M, Gursky LC, Faveri M, Tsuzuki CO, Figueiredo LC. J Clin Periodontol. 2009;36(10):857-67. doi: 10.1111/j.1600-051X.2009.01471.x
185	Quantification of periodontal pathogens by paper point sampling from the coronal and apical aspect of periodontal lesions by real-time PCR.<br>Jervøe-Storm PM, AlAhdab H, Koltzscher M, Fimmers R, Jepsen S. Clin Oral Investig. 2010;14(5):533-41. doi: 10.1007/s00784-009-0333-x
186	Full-mouth ultrasonic debridement associated with amoxicillin and metronidazole in the treatment of severe chronic periodontitis.<br>Ribeiro Edel P, Bittencourt S, Zanin IC, Bovi Ambrosano GM, Sallum EA, Nociti FH, Gonçalves RB, Casati MZ. J Periodontol. 2009;80(8):1254-64. doi: 10.1902/jop.2009.080403
187	Probiotic effects of orally administered Lactobacillus salivarius WB21-containing tablets on periodontopathic bacteria: a double-blinded, placebo-controlled, randomized clinical trial.<br>Mayanagi G, Kimura M, Nakaya S, Hirata H, Sakamoto M, Benno Y, Shimauchi H. J Clin Periodontol. 2009;36(6):506-13. doi: 10.1111/j.1600-051X.2009.01392.x
188	Local application of tetracycline solution with a microbrush: an alternative treatment for persistent periodontitis.<br>Bosco JMD, Lopes BMV, Bosco AF, Spolidorio DMP, Marcantonio RAC. Quint Int. 2009;40(1):29-40.
190	Moxifloxacin as an adjunctive antibiotic in the treatment of severe chronic periodontitis.<br>Guentsch A, Jentsch H, Pfister W, Hoffmann T, Eick S. J Periodontol. 2008;79(10):1894-903. doi: 10.1902/jop.2008.070493
191	Periodontal bacterial profiles in pregnant women: response to treatment and associations with birth outcomes in the obstetrics and periodontal therapy (OPT) study.<br>Novak MJ, Novak KF, Hodges JS, Kirakodu S, Govindaswami M, Diangelis A, Buchanan W, Papapanou PN, Michalowicz BS. J Periodontol. 2008;79(10):1870-9. doi: 10.1902/jop.2008.070554
193	Photodynamic therapy as an adjunct to non-surgical periodontal treatment: a randomized, controlled clinical trial.<br>Christodoulides N, Nikolidakis D, Chondros P, Becker J, Schwarz F, Rössler R, Sculean A. J Periodontol. 2008;79(9):1638-44. doi: 10.1902/jop.2008.070652
194	Nutritional intervention in patients with periodontal disease: clinical, immunological and microbiological variables during 12 months.<br>Jenzsch A, Eick S, Rassoul F, Purschwitz R, Jentsch H. Br J Nutr. 2009;101(6):879-85. doi: 10.1017/S0007114508047776
195	Periodontal debridement as a therapeutic approach for severe chronic periodontitis: a clinical, microbiological and immunological study.<br>Del Peloso Ribeiro E, Bittencourt S, Sallum EA, Nociti FH Jr, Gonçalves RB, Casati MZ. J Clin Periodontol. 2008;35(9):789-98. doi: 10.1111/j.1600-051X.2008.01292.x
197	Photodynamic therapy as adjunct to non-surgical periodontal treatment in patients on periodontal maintenance: a randomized controlled clinical trial.<br>Chondros P, Nikolidakis D, Christodoulides N, Rössler R, Gutknecht N, Sculean A. Lasers Med Sci. 2009;24(5):681-8. doi: 10.1007/s10103-008-0565-z
198	Microbial changes in patients with acute periodontal abscess after treatment detected by PadoTest.<br>Eguchi T, Koshy G, Umeda M, Iwanami T, Suga J, Nomura Y, Kawanami M, Ishikawa I. Oral Dis. 2008;14(2):180-4. doi: 10.1111/j.1601-0825.2007.01370.x
199	Clinical and microbiological analysis of subjects treated with Brånemark or AstraTech implants: a 7-year follow-up study.<br>Renvert S, Lindahl C, Renvert H, Persson GR. Clin Oral Implants Res. 2008;19(4):342-7. doi: 10.1111/j.1600-0501.2007.01476.x
202	Subantimicrobial dose doxycycline effects on osteopenic bone loss: microbiologic results.<br>Walker C, Puumala S, Golub LM, Stoner JA, Reinhardt RA, Lee HM, Payne JB. J Periodontol. 2007;78(8):1590-601. doi: 10.1902/jop.2007.070015
203	Minocycline HCl microspheres reduce red-complex bacteria in periodontal disease therapy.<br>Goodson JM, Gunsolley JC, Grossi SG, Bland PS, Otomo-Corgel J, Doherty F, Comiskey J. J Periodontol. 2007;78(8):1568-79. doi: 10.1902/jop.2007.060488
204	Microbiological findings after periodontal therapy using curettes, Er:YAG laser, sonic, and ultrasonic scalers.<br>Derdilopoulou FV, Nonhoff J, Neumann K, Kielbassa AM. J Clin Periodontol. 2007;34(7):588-98. doi: 10.1111/j.1600-051X.2007.01093.x
205	Effects of full-mouth scaling and root planing in conjunction with systemically administered azithromycin.<br>Gomi K, Yashima A, Nagano T, Kanazashi M, Maeda N, Arai T. J Periodontol. 2007;78(3):422-9. doi: 10.1902/jop.2007.060247
206	Microbiological outcomes of quadrant versus full-mouth root planing as monitored by real-time PCR.<br>Jervøe-Storm PM, AlAhdab H, Semaan E, Fimmers R, Jepsen S. J Clin Periodontol. 2007;34(2):156-63. doi: 10.1111/j.1600-051X.2006.01035.x
207	Periodontal healing after non-surgical therapy with a new ultrasonic device: a randomized controlled clinical trial.<br>Christgau M, Männer T, Beuer S, Hiller KA, Schmalz G. J Clin Periodontol. 2007;34(2):137-47. doi: 10.1111/j.1600-051X.2006.01031.x
209	Periodontal healing after non-surgical therapy with a modified sonic scaler: a controlled clinical trial.<br>Christgau M, Männer T, Beuer S, Hiller KA, Schmalz G. J Clin Periodontol. 2006;33(10):749-58. doi: 10.1111/j.1600-051X.2006.00981.x

210	Effects of metronidazole plus amoxicillin as the only therapy on the microbiological and clinical parameters of untreated chronic periodontitis.<br>López NJ, Socransky SS, Da Silva I, Japlit MR, Haffajee AD. J Clin Periodontol. 2006;33(9):648-60. doi: 10.1111/j.1600-051X.2006.00957.x
211	Short-term clinical and microbiologic effects of pocket debridement with an Er:YAG laser during periodontal maintenance.<br>Tomasi C, Schander K, Dahlén G, Wennström JL. J Periodontol. 2006;77(1):111-8. doi: 10.1902/jop.2006.77.1.111
212	Local oxygen therapy for treating acute necrotizing periodontal disease in smokers.<br>Gaggl AJ, Rainer H, Grund E, Chiari FM. J Periodontol. 2006;77(1):31-8. doi: 10.1902/jop.2006.77.1.31
213	Clinical and microbiological effects of different antimicrobials on generalized aggressive periodontitis.<br>Xajigeorgiou C, Sakellari D, Slini T, Baka A, Konstantinidis A. J Clin Periodontol. 2006;33(4):254-64. doi: 10.1111/j.1600-051X.2006.00905.x
216	Dynamics of initial subgingival colonization of 'pristine' peri-implant pockets.<br>Quirynen M, Vogels R, Peeters W, van Steenberghe D, Naert I, Haffajee A. Clin Oral Implants Res. 2006;17(1):25-37. doi: 10.1111/j.1600-0501.2005.01194.x
217	Supportive periodontal therapy using mechanical instrumentation or 2% minocycline gel: a 12 month randomized, controlled, single masked pilot study.<br>McColl E, Patel K, Dahlen G, Tonetti M, Graziani F, Suvan J, Laurell L. J Clin Periodontol. 2006;33(2):141-50. doi: 10.1111/j.1600-051X.2005.00879.x
218	Microbial colonization patterns predict the outcomes of surgical treatment of intrabony defects.<br>Heitz-Mayfield L, Tonetti MS, Cortellini P, Lang NP; European Research Group on Periodontology (ERGOPERIO). J Clin Periodontol. 2006;33(1):62-8. doi: 10.1111/j.1600-051X.2005.00872.x
227	Antibiotic resistance profile of the subgingival microbiota following systemic or local tetracycline therapy.<br>Rodrigues RM, Gonçalves C, Souto R, Feres-Filho EJ, Uzeda M, Colombo AP. J Clin Periodontol. 2004;31(6):420-7. doi: 10.1111/j.1600-051X.2004.00493.x
230	Quadrant root planing versus same-day full-mouth root planing. II. Microbiological findings.<br>Apatzidou DA, Riggio MP, Kinane DF. J Clin Periodontol. 2004;31(2):141-8. doi: 10.1111/j.0303-6979.2004.00462.x
231	Debridement and local application of tetracycline-loaded fibres in the management of persistent periodontitis: results after 12 months.<br>Aimetti M, Romano F, Torta I, Cirillo D, Caposio P, Romagnoli R. J Clin Periodontol. 2004;31(3):166-72. doi: 10.1111/j.0303-6979.2004.00457.x
232	Relationship between periodontal pocket sulfide levels and subgingival species.<br>Torresyap G, Haffajee AD, Uzel NG, Socransky SS. J Clin Periodontol. 2003;30(11):1003-10. doi: 10.1034/j.1600-051x.2003.00377.x
235	The effect of a triclosan-containing dentifrice on the progression of periodontal disease in an adult population.<br>Cullinan MP, Westerman B, Hamlet SM, Palmer JE, Faddy MJ, Seymour GJ. J Clin Periodontol. 2003;30(5):414-9. doi: 10.1034/j.1600-051x.2003.20030.x
237	A split-mouth study on periodontal and microbial parameters in children with complete unilateral cleft lip and palate.<br>Quirynen M, Dewinter G, Avontroodt P, Heidbüchel K, Verdonck A, Carels C. J Clin Periodontol. 2003;30(1):49-56. doi: 10.1034/j.1600-051x.2003.300108.x
244	Guided tissue regeneration in intrabony defects using an experimental bioresorbable polydioxanon (PDS) membrane. A 24-month split-mouth study.<br>Christgau M, Bader N, Felden A, Gradl J, Wenzel A, Schmalz G. J Clin Periodontol. 2002;29(8):710-23. doi: 10.1034/j.1600-051x.2002.290808.x
245	Initial effect of controlled release chlorhexidine on subgingival microorganisms.<br>Daneshmand N, Jorgensen MG, Nowzari H, Morrison JL, Slots J. J Periodontal Res. 2002;37(5):375-9. doi: 10.1034/j.1600-0765.2002.01003.x
246	Local antimicrobial therapy after initial periodontal treatment.<br>Salvi GE, Mombelli A, Mayfield L, Rutar A, Suvan J, Garrett S, Lang NP. J Clin Periodontol. 2002;29(6):540-50. doi: 10.1034/j.1600-051x.2002.290611.x
250	Adjunctive effects to non-surgical periodontal therapy of systemic metronidazole and amoxycillin alone and combined. A placebo controlled study.<br>Rooney J, Wade WG, Sprague SV, Newcombe RG, Addy M. J Clin Periodontol. 2002;29(4):342-50. doi: 10.1034/j.1600-051x.2002.290410.x
256	One-stage full-mouth disinfection. Long-term microbiological results analyzed by checkerboard DNA-DNA hybridization.<br>De Soete M, Mongardini C, Peuwels M, Haffajee A, Socransky S, van Steenberghe D, Quirynen M. J Periodontol. 2001;72(3):374-82. doi: 10.1902/jop.2001.72.3.374
259	A 2-step non-surgical procedure and systemic antibiotics in the treatment of rapidly progressive periodontitis.<br>Sigusch B, Beier M, Klinger G, Pfister W, Glockmann E. J Periodontol. 2001;72(3):275-83. doi: 10.1902/jop.2001.72.3.275
260	Amoxicillin plus metronidazole in the treatment of adult periodontitis patients. A double-blind placebo-controlled study.<br>Winkel EG, Van Winkelhoff AJ, Timmerman MF, Van der Velden U, Van der Weijden GA. J Clin Periodontol. 2001;28(4):296-305. doi: 10.1034/j.1600-051x.2001.028004296.x
262	Treatment with subantimicrobial dose doxycycline improves the efficacy of scaling and root planing in patients with adult periodontitis.

	Caton JG, Ciancio SG, Blieden TM, Bradshaw M, Crout RJ, Hefti AF, Massaro JM, Polson AM, Thomas J, Walker C. J Periodontol. 2000;71(4):521-32. doi: 10.1902/jop.2000.71.4.521
263	Systemic doxycycline administration in the treatment of periodontal infections (II). Effect on antibiotic resistance of subgingival species.<br>Feres M, Haffajee AD, Goncalves C, Allard KA, Som S, Smith C, Goodson JM, Socransky SS. J Clin Periodontol. 1999;26(12):784-92. doi: 10.1111/j.1600-051x.1999.tb02521.x
264	Systemic doxycycline administration in the treatment of periodontal infections (I). Effect on the subgingival microbiota.<br>Feres M, Haffajee AD, Goncalves C, Allard KA, Som S, Smith C, Goodson JM, Socransky SS. J Clin Periodontol. 1999;26(12):775-83. doi: 10.1111/j.1600-051x.1999.tb02520.x
265	Repeated local metronidazole-therapy as adjunct to scaling and root planing in maintenance patients.<br>Riep B, Purucker P, Bernimoulin JP. J Clin Periodontol. 1999;26(11):710-5. doi: 10.1034/j.1600-051x.1999.t01-2-261101.x
266	Clonal infection with Actinobacillus actinomycetemcomitans following periodontal therapy.<br>Ehmke B, Schmidt H, Beikler T, Kopp C, Karch H, Klaiber B, Flemmig TF. J Dent Res. 1999;78(9):1518-24. doi: 10.1177/00220345990780090601
267	Longitudinal effect of non-surgical treatment and systemic metronidazole for 1 week in smokers and non-smokers with refractory periodontitis: a 5-year study.<br>Söder B, Nedlich U, Jin LJ. J Periodontol. 1999;70(7):761-71. doi: 10.1902/jop.1999.70.7.761
268	Clinical and microbiological effects of initial periodontal therapy in conjunction with amoxicillin and clavulanic acid in patients with adult periodontitis. A randomised double-blind, placebo-controlled study.<br>Winkel EG, van Winkelhoff AJ, Barendregt DS, van der Weijden GA, Timmerman MF, van der Velden U. J Clin Periodontol. 1999;26(7):461-8. doi: 10.1034/j.1600-051x.1999.260708.x
269	A 15-month evaluation of the effects of repeated subgingival minocycline in chronic adult periodontitis.<br>van Steenberghe D, Rosling B, Söder PO, Landry RG, van der Velden U, Timmerman MF, McCarthy EF, Vandenhoven G, Wouters C, Wilson M, Matthews J, Newman HN. J Periodontol. 1999;70(6):657-67. doi: 10.1902/jop.1999.70.6.657
270	One stage full- versus partial-mouth disinfection in the treatment of chronic adult or generalized early-onset periodontitis. II. Long-term impact on microbial load.<br>Quirynen M, Mongardini C, Pauwels M, Bollen CM, Van Eldere J, van Steenberghe D. J Periodontol. 1999;70(6):646-56. doi: 10.1902/jop.1999.70.6.646
271	Microbiological and clinical effects of a 1% chlorhexidine-gel in untreated periodontal pockets from adult periodontitis patients.<br>Piccolomini R, Di Bonaventura G, Catamo G, Tumini V, Di Placido G, D'Ercole S, Perfetti G, Paolantonio M. New Microbiol. 1999;22(2):111-6.
273	The prevalence of Actinobacillus actinomycetemcomitans, Porphyromonas gingivalis, and Bacteroides forsythus in humans 1 year after 4 randomized treatment modalities.<br>Shiloah J, Patters MR, Dean JW 3rd, Bland P, Toledo G. J Periodontol. 1998;69(12):1364-72. doi: 10.1902/jop.1998.69.12.1364
274	Clinical and microbiological effects of adjunctive antibiotics in treatment of localized juvenile periodontitis. A controlled clinical trial.<br>Tinoco EM, Beldi MI, Campedelli F, Lana M, Loureiro CA, Bellini HT, Rams TE, Tinoco NM, Gjermo P, Preus HR. J Periodontol. 1998;69(12):1355-63. doi: 10.1902/jop.1998.69.12.1355
276	Additional clinical and microbiological effects of amoxicillin and metronidazole after initial periodontal therapy.<br>Winkel EG, van Winkelhoff AJ, van der Velden U. J Clin Periodontol. 1998;25(11 Pt 1):857-64. doi: 10.1111/j.1600-051x.1998.tb02382.x
277	Local metronidazole application in maintenance patients. Clinical and microbiological evaluation.<br>Rudhart A, Purucker P, Kage A, Hopfenmüller W, Bernimoulin JP. J Periodontol. 1998;69(10):1148-54. doi: 10.1902/jop.1998.69.10.1148
278	Effects of topical metronidazole and tetracycline in treatment of adult periodontitis.<br>Lie T, Bruun G, Böe OE. J Periodontol. 1998;69(7):819-27. doi: 10.1902/jop.1998.69.7.819
280	Treatment of periodontal pockets with a diode laser.<br>Moritz A, Schoop U, Goharkhay K, Schauer P, Doertbudak O, Wernisch J, Sperr W. Lasers Surg Med. 1998;22(5):302-11. doi: 10.1002/(sici)1096-9101(1998)22:5<302::aid-lsm7>3.0.co;2-t
281	The use of metronidazole and amoxicillin in the treatment of advanced periodontal disease. A prospective, controlled clinical trial.<br>Berglundh T, Krok L, Liljenberg B, Westfelt E, Serino G, Lindhe J. J Clin Periodontol. 1998;25(5):354-62. doi: 10.1111/j.1600-051x.1998.tb02455.x
282	The effect of a 1-stage full-mouth disinfection on oral malodor and microbial colonization of the tongue in periodontitis. A pilot study.<br>Quirynen M, Mongardini C, van Steenberghe D. J Periodontol. 1998;69(3):374-82. doi: 10.1902/jop.1998.69.3.374
283	The effect of a one-stage full-mouth disinfection on different intra-oral niches. Clinical and microbiological observations.<br>Bollen CM, Mongardini C, Papaioannou W, Van Steenberghe D, Quirynen M. J Clin Periodontol. 1998;25(1):56-66. doi: 10.1111/j.1600-051x.1998.tb02364.x
